# Sulfur Oxygenase Reductase (Sor) in the Moderately Thermoacidophilic Leaching Bacteria: Studies in *Sulfobacillus thermosulfidooxidans* and *Acidithiobacillus caldus*

**DOI:** 10.3390/microorganisms3040707

**Published:** 2015-10-21

**Authors:** Claudia Janosch, Francisco Remonsellez, Wolfgang Sand, Mario Vera

**Affiliations:** 1 Biofilm Centre, Aquatische Biotechnologie, Universität Duisburg-Essen, Universitätstraße 5, Essen, 45141, Germany; E-Mails: claudia.janosch@gmx.de (C.J.); wolfgang.sand@uni-due.de (W.S.); 2 Laboratorio de Tecnología de Membranas, Biotecnología y Medio Ambiente, Departamento de Ingeniería Química, Universidad Católica del Norte, Antofagasta 1270709, Chile; E-Mail: fremonse@ucn.cl

**Keywords:** *Sulfobacillus thermosulfidooxidans*, *Acidithiobacillus caldus*, sulfur metabolism, sulfur oxygenase reductase, genome

## Abstract

The sulfur oxygenase reductase (Sor) catalyzes the oxygen dependent disproportionation of elemental sulfur, producing sulfite, thiosulfate and sulfide. Being considered an “archaeal like” enzyme, it is also encoded in the genomes of some acidophilic leaching bacteria such as *Acidithiobacillus caldus*, *Acidithiobacillus thiooxidans*, *Acidithiobacillus ferrivorans* and *Sulfobacillus thermosulfidooxidans*, among others. We measured Sor activity in crude extracts from *Sb. thermosulfidooxidans* DSM 9293^T^. The optimum temperature for its oxygenase activity was achieved at 75 °C, confirming the “thermophilic” nature of this enzyme. Additionally, a search for genes probably involved in sulfur metabolism in the genome sequence of *Sb. thermosulfidooxidans* DSM 9293^T^ was done. Interestingly, no sox genes were found. Two *sor* genes, a complete heterodisulfidereductase (*hdr*) gene cluster, three tetrathionate hydrolase (*tth*) genes, three sulfide quinonereductase (*sqr*), as well as the *doxD* component of a thiosulfate quinonereductase (*tqo*) were found. Seven *At. caldus* strains were tested for Sor activity, which was not detected in any of them. We provide evidence that an earlier reported Sor activity from *At. caldus* S1 and S2 strains most likely was due to the presence of a *Sulfobacillus* contaminant.

## 1. Introduction

Control of biological sulfur oxidation is important in bioleaching operations for the industrial bioleaching of metal sulfides or heavy metal recovery from industrial wastes [1]. Moderately thermoacidophilic leaching bacteria such as *At. caldus* and *Sb. thermosulfidooxidans*, are frequently found in leaching operations [2,3]. *Sulfobacillus* are Gram-positive, rod shaped, spore forming bacteria able to use S°, reduced inorganic sulfur compounds (RISC), ferrous iron and/or metal sulfide minerals as energy sources under chemolithoautotrophic or mixotrophic conditions [4]. Heterotrophic growth is also possible under low concentrations of organic substrates [5]. Complete genome sequences for *Sb. thermosulfidooxidans* DSM 9293^T^ (NCBI taxon: 929705) and “Cutipay” strain [6], as well as for *Sb. acidophilus* strains DSM 10332^T^ [7] and TPY [8] are available. *At. caldus* is a moderately thermophilic acidophilic Gram-negative bacterium able to chemolithoautotrophically oxidize S° and RISC such as tetrathionate or thiosulfate [9]. Although it cannot oxidize ferrous iron or pyrite, it can grow on RISCs resulting from pyrite oxidation in combination with iron oxidizers like *Leptospirillum* spp. [10]. It is also able to oxidize arsenopyrite [11]. Complete genome sequences exist for *At. caldus* DSM 8584^T^ [12] and *At. caldus* SM-1[13].

In thermophilic archaea, such as *Acidianus ambivalens*, a sulfur oxygenase reductase (Sor) catalyzes an oxygen-dependent S° disproportionation reaction to thiosulfate, sulfite and hydrogen sulfide. In this one 3 moles of S° are converted into 1 mole sulfite and 2 moles of hydrogen sulfide [14,15]. It does not require addition of external cofactors for its activity. Although no energy conservation occurs during Sor catalysis, its reaction products can be further oxidized by other enzymes to sulfate [16]. Recombinant Sor enzymes from *A. ambivalens* [17], *Acidianus tengchongensis* [18], *Aquifex aeolicus* [19] and *Halothiobacillus neapolitanus* [20] have been expressed in *E. coli* and their activity has been reported.

Several other enzymes for sulfur oxidation are conserved among thermophilic and mesophilic acidophiles. Sulfite is substrate for sulfite acceptor oxidoreductases (Saor), which catalyze its oxidation to sulfate [21]. In addition sulfite can abiotically react with an excess of S° to form thiosulfate, which is substrate for a thiosulfate:quinone oxidoreductase (Tqo), catalyzing the generation of tetrathionate and feeding the electrons into the quinone pool in the cytoplasmic membrane. *A. ambivalens* Tqo is composed of two subunits, named DoxD and DoxA [22]. Interestingly, in *At. ferrooxidans* both *dox* genes are fused and duplicated, named *doxDA1* and *doxDA2* [23]. *At. caldus* Dox proteins have similar sizes as *At. ferrooxidans* DoxDA, but just the DoxD domain is found to be present [24]. In addition to its generation by the abiotic reaction of S° with sulfite, thiosulfate is generated by the reaction of tetrathionate hydrolase (Tth), which in *A. ambivalens* catalyzes its decomposition to sulfate, thiosulfate and S° [16]. The *Acidianus* Tth is biochemically and phylogenetically similar to the *Acidithiobacillus* Tth [25]. Both enzymes are located outside the cell and have optimal activities at acidic pH [26].

Apart from the Saor activity, the enzymes adenylylsulfate (Aps) reductase and adenylyl transferase (Apat) are involved in the generation of ATP from sulfite by substrate level phosphorylation [27]. The third product, hydrogen sulfide, is oxidized back to S° by the membrane bound sulfide:quinone oxidoreductase (Sqr) [28,29,30]. All electrons made available from sulfur oxidation in the course of Sqr, Sar and Tqo activities reduce *Caldariella* quinones (CQ) but not cytochromes [16,22]. The bacterial “Sox” (sulfur-oxidizing) system consists of a set of dehydrogenases and other periplasmic proteins which catalyze the oxidation of sulfide, S°, thiosulfate and sulfite to sulfate, accompanied by subsequent electron transfers through di- and mono-heme cytochromes [31]. The *sox* gene cluster of *Paracoccus pantotrophus* comprises 15 genes, encoding among others for the periplasmic proteins SoxXA, SoxYZ, SoxB, Sox(CD)_2_, which interact with each other [32,33]. SoxXA is composed by the diheme citochrome SoxA and the monoheme cytochrome SoxX. The SoxYZ complex does not contain cofactors and is probably involved in the reaction cycle. Sox(CD)_2_ is composed of the molybdoprotein SoxC and the diheme cytochrome C protein SoxD. An incomplete *sox* system, where *soxCD* orthologous genes are missing, has been found encoded in the genomes of *At. caldus* [12] and *Acidithiobacillus ferrivorans* [34]. Enzyme reconstitution assays with *P. pantotrophus* Sox, have shown that the absence of the tetrameric protein Sox(CD)_2_ reduced the activity of the Sox pathway from 8 mol of electrons/mol of thiosulfate to two mols of electrons/mol of thiosulfate [35]. The Sox multienzyme-complex is absent in the mesophilic acidophilic leaching bacterium *At. ferrooxidans* [36], in which a sulfur dioxygenase (Sdo) has been proposed to be responsible for the S° oxidation step [37]. Recently a deletion mutant strain of a putative *At. ferrooxidans* ATCC 23270^T^ Sdo was constructed. The mutant strain still possessed Sdo activity, suggesting a dissimilatory function of this enzyme and the presence of other enzyme(s) responsible for Sdo activity [38]. By bioinformatics and transcriptomic analyses it has also been suggested that the gene cluster *hdrABC* (heterodisulfide reductase) and some of its accessory proteins, which are conserved in several acidithiobacilli as well as in sulfur oxidizing archaea, could catalyze a similar sulfur oxidation reaction as Sdo [36]. However, biochemical evidence to support this proposal is missing. Proteins containing Rhodanese domain(s) are ubiquitous sulfur transferase enzymes that catalyze the transfer of a sulfane sulfur atom from a donor to an appropriate sulfur acceptor *in vitro*. These can belong to the thiosulfate:cyanide sulfurtransferase (TST) or the 3-mercaptopyruvate sulfurtransferases (MSTs) family [39].

*At. caldus* ATCC 53993^T^ possesses a *sor* gene encoded on its genome sequence [12]. Previously we reported the presence of Sor enzyme activity in *At. caldus* strains S1 and S2 [40]. However, our attempts to measure Sor activity in *At. caldus*^T^ and some other strains were unsuccessful (see Materials and Methods). Four *sor* sequences were obtained from metagenomic DNA samples from a bioreactor treating gold concentrates. This reactor contained species of *Leptospirillum*, *Sulfobacillus*, *Acidithiobacillus* and *Sphingomonas*. One of these *sor* genes (DQ480734) was cloned and expressed in *E. coli.* The recombinant Sor showed an optimal oxygenase activity of 3.76 U/mg at 75–80 °C and pH 7.5. This protein was attributed to belong to *At. caldus* SM-1 [41]. However, further analysis of the complete genome sequence of *At. caldus* SM-1 showed that the *sor* gene was missing in this strain. Its deletion was explained by an event of transposition of the element ISAtc1 [13].

BLAST searches revealed the presence of *sor* genes encoded in the genomes of *Sb. thermosulfidooxidans* DSM 9293^T^ and in *S. acidophilus* strains TPY and DSM 10332^T^. In this article we report that *Sb. thermosulfidooxidans* DSM 9293^T^ crude extracts possess Sor activity. We also provide evidence that the previously reported Sor activity in *At. caldus* strains S1 and S2 was most likely due to the presence of a *Sulfobacillus* contaminant in strains S1 and S2.

## 2. Materials and Methods

### 2.1. Strains Used in This Study

*Sb. thermosulfidooxidans* DSM 9232^T^, the *At. caldus* strains: DSM 8584^T^, DSM 9466 (former C-SH12), S1, S2, MNG, f, and #6 were used. Strains S1 and S2 were provided by Zhou H. (Central South University of Changsha, China). Strains MNG, f, & 6# were described by Rawlings *et al.* [42].

All *A. caldus* strains as well as *Sulfolobus metallicus* DSM 6482^T^ were grown in Mackintosh (Mac) basal salt medium [43], at pH 2.5, supplemented with 5 g/L S° and traces of ferric sulfate (~1 mg/L). Media were autoclaved at 110 °C for 90 min. *S. metallicus* was used as a positive control for Sor enzyme activity tests. For *Sb. thermosulfidooxidans* and *S. metallicus*, 0.2 g/L yeast extract was added after autoclaving. Batch cultures (10 L) of *Sb. thermosulfidooxidans* or *At. caldus* for Sor enzyme assays were grown at 45 °C with aeration and stirring at 300 rpm. *S. metallicus* cultures (5L) were incubated at 65 °C without shaking. When necessary, S° was removed by low speed centrifugation for 5 min at 120× *g* before cell harvesting. Additionally, *Sb. thermosulfidooxidans* was also grown in Mac basal salt solution with 2 g/L ferrous iron ions (supplied as FeSO_4_·7 H_2_O) and 0.2 g/L yeast extract). During our experiments, after detecting *Sulfobacillus* contamination, *At. caldus* strains S1 and S2 were repurified by three consecutive rounds of maximal serial 10-fold dilutions in Mac medium amended with S°.

### 2.2. Molecular Biology Techniques

DNA was extracted as described [44]. PCR reactions were done in a final reaction volume of 25 μL using 20–50 ng of genomic DNA template, 1× Green Flexi buffer, 2.5 mM MgCl_2_, 1 mM dNTPs, 10 pmol of each single primer and 0.5 U GoTaq^®^ DNA polymerase (Promega^®^, MI, Wisconsin, USA). Reactions were incubated in an Eppendorf Mastercyler 5332, (Hamburg, Germany). The following temperature program was used: five minutes initial denaturation at 95 °C followed by 30 to 40 cycles of denaturation for 30 s at 95 °C, primer annealing for 30 to 45 s at 50 to 58 °C, depending on each primer pair used (Table 1), and 0.5 to 1.5 min of extension at 72 °C, depending on the size of the expected amplicon. A final extension step was done for 3 min at 72 °C.

Purity tests of *At. caldus* S1 and S2 strains were done by a two-stage nested polymerase chain reaction (PCR)-mediated detection method [45]. Additionally, *At. caldus* strains S1 and S2 were tested for archaeal contamination [46,47]. *At. caldus sor* genes were amplified with consensus-degenerate hybrid oligonucleotide primer (CODEHOP)-PCR primers [48]. For this, the primer pairs PCJ2_for-PCJ3_rev and bsor_1F-bsor_2R (Table 1) were designed based on the alignments of amino acid sequences of all Sor proteins, or just the bacterial ones available at the time of this study, respectively. The latter ones included *At. caldus*^T^ (EET26704.1), uncultured bacterium BSB (gi:94470458), *Halothiobacillus neapolitanus* C2 (ACX96058.1) and *Desulfomicrobium baculatum* DSM 4028 (ACU89275.1). Positive *sor* gene amplicons were cloned using the pGEM^®^-T vector system (Promega^®^). Ligation reactions were transformed in competent *E. coli* DH5α cells. Plasmids were isolated using Roti^®^-Prep Plasmid MINI Kit (Carl Roth, Karlsruhe, Germany). The presence of a cloned insert was confirmed by PCR using T7 and SP6 primers, adjacent to its cloning site. DNA sequencing was done in “Zentraler DNA-Sequenzierservice”, Universitätsklinikum Essen.

**Table 1:** Polymerase chain reaction (PCR) primers used in this study.

Primer	Sequence 5ʹ→3ʹ	Target Gene	Amplicon Size	References
16s_27fw	agagtttgatcctggctcag	16S rDNA	~1.5 kb	Lane *et al.* 1991 [46]
16s_1492rv	gcctaccttgttacgactt	Bacteria		
Arch25F	cyggttgatcctgccrg	18S rDNA	~1.5 kb	Achenbach and Woese 1995 [47]
Arch1492R	tacggytaccttgttacgactt	Archaea		
sorC1-F	Gtiggiccnaargtntgy *	*Sor*	~230 bp	Chen *et al.* 2007 [41]
sorH1-R	rtgcatntcytcrtgrtc			
bsor_1F	gtccttcgagaccatgatgmargtnggncc	bacterial*sor (CODEHOP)*	~800 bp	This study
bsor_2R	ccgccactgggcctsytccatcatng			
PCJ2_for	caggcctcccagcaggtnggnccnaa	*sor*(CODEHOP)	840 bp	This study
PCJ3_rev	ctcccgccatgaggtgtcctccatnayngg			
SULFO170F	caatcccgcatacgttcc	16S rDNA	436 bp	De Wulf-Durand *et al.* 1997 [45]
SULFO606R	aaaccgctacgtatcgcac	*Sulfobacillus* spp.		
CALD460F	atccgaatacggtctgcta	16S rDNA	~1 kb	De Wulf-Durand *et al.* 1997 [45]
CALD1475R	tataccgtggtcgtcgcc	*At. caldus*		
THIO458F	gggtgctaatawcgcctgctg	16S rDNA	~1 kb	De Wulf-Durand *et al.* 1997 [45]
THIO1473R	taccgtggtcatcgccct	*At. thiooxidans*		
LEPTO176F	cgaatagtatccggttccg	16S rDNA	503 bp	De Wulf-Durand *et al.* 1997 [45]
LEPTO679R	aaattccgcttccctctcc	*Leptospirillum*spp.		
FERRO458F	gggttctaatacaatctgct	16S rDNA	~1 kb	De Wulf-Durand *et al.* 1997 [45]
FERRO1473	taccgtggtaaccgccct	*At. ferrooxidans*		
T7	taatacgactcactataggg	Promoterregions	158 bp	Promega® pGEM-T vector manual
SP6	atttaggtgacactatagaa	in pGEM®-T vector		

* i, inosine.

### 2.3. Bioinformatics and Phylogeny Analyses

Gene sequences were analyzed in the databases of the National Center for Biotechnology Information NCBI (www.ncbi.nlm.nih.gov), the Kyoto Encyclopedia of Genes and Genomes (KEGG) (www.genome.jp/kegg/) and the DOE Joint Genome Institute (JGI) (https://signon.jgi.doe.gov/), in which genome sequences are available upon registration. To compare gene or protein sequences, multiple sequence alignments with Clustal W (www.ebi.ac.uk/Tools/msa/clustalw2/) were done [49]. All Sor sequences found after BLAST searches in NCBI & JGI databases at the time of this study were used. Additionally, the *At. caldus* Sor sequences obtained in this study as well as the four clones (DQ480731-DQ480734) containing Sor sequences previously attributed to *At. caldus* SM-1 [41] were included. For phylogenetic analysis, sequences were aligned using the multiple sequence comparison by log-expectation (MUSCLE) tool [50] and a maximum likelihood analysis with the substitution model (WAG) was conducted. Support was evaluated using 100 bootstrap replications. The phylogenetic tree was edited using MEGA5 [51].

### 2.4. Cell Harvest and Preparation of Cell-Free Extracts

Ten liters of batch cultures were harvested by centrifugation at 8700× *g* for 10 min. After removal of S°, cells were pelleted at 8700× *g* for 10 min and washed twice with a solution containing 2 mM NH_4_Cl, 0.1 mM MgCl_2_, 1 mM CaCl_2_, pH 3 [37]. Cell pellets were resuspended at 1/10 (*w*/*v*) in 100 mM Tris-HCl, pH 7.5. Afterwards, cells were broken using a French^®^ Press (Thermo Electron Corporation; French Pressure Cell Press, Milford, MA, USA) in four passages of 10–15 mL. Crude extracts were dispatched in 2 mL aliquots and centrifuged at 20,800× *g* for 20 min at 4 °C. Supernatants were combined and protein concentrations were measured as described [52].

### 2.5. Sor Enzyme Assays

Sor enzyme assays for *Sb. thermosulfidooxidans* were performed aerobically at 45 °C and from 65 °C to 80 °C (in 5 °C intervals). Reaction mixtures (25 mL) contained 20 mL of “dispersed elemental S°” [37] and 5 mL of crude extracts (0.2 mg/mL protein) in 100 mM Tris-HCl, pH 7.5. Supelco glass serum bottles of 43 mm by 73 mm (Sigma-Aldrich, Darmstadt, Germany) were used. Immediately after mixing, bottles were closed with rubber lids (Butyl septum; Ochs GmbH, Bovenden, Germany) in order to avoid hydrogen sulfide loss. Bottles were stirred at 180 rpm during enzyme measurements. Under our assay conditions, Sor activity was tested with ~0.04 mg/mL total protein and ~17 mM dispersed S°. Samples (1.5 mL) were taken off with a syringe after 1 min, from 5 to 30 min (in 5 min intervals) and at 40 min. These samples were immediately filtered through nylon filters (Rotilabo^®^-Spritzenfilter 0.45 µm, Carl Roth, Karlsruhe, Germany). Additionally, for the determination of the reductase activity (sulfide production) 200 µL of these samples were fixed with addition of 200 µL of 2% *w*/*v* Zn-acetate. The sum of sulfite, sulfate and thiosulfate, as equivalent for oxygenase activity, was quantified by ion-exchange chromatography as further described. Specific activities were calculated from the linear increase of the reaction products. One Unit (U) of enzyme activity was defined as 1 μmol of formed sulfite, sulfate and thiosulfate (oxygenase) or hydrogen sulfide (reductase) per min per mg of protein. Optimum pH values for *Sb. thermosulfidooxidans* Sor activity were determined at 75 °C between pH 6.5–8.5 (in 0.5 pH steps). The optimum temperature of Sor activity was determined in the range of 65 °C–80 °C (in 5 °C steps) at pH 7.5. To determine non-enzymatic reactions, control assays with addition of 40 mg/L Bovine Serum Albumin (Sigma^®^) were done. These values were subtracted from the assays with crude extracts. Sor enzyme assays for *At. caldus* strains were done as mentioned at 45 °C and 65 °C at pH 7.5. Additionally, Sor activity of *S. metallicus* was measured at pH 8 and 65 °C as positive control.

### 2.6. Determination of Thiosulfate, Sulfite, Sulfate and Sulfide

Thiosulfate, sulfite and sulfate were quantified by ion-exchange chromatography and conductivity detection as described ([53] Schippers, 2002 #789). The DIONEX system DX-500 (Thermo Scientific, USA) G with an AS 3500 autosampler, ASRD ULTRA II 2 mm suppressor, conductivity detector CD20,gradient generator EG 50 in combination with the EluGen cartridge EGC II KOH (Thermo Scientific, USA), guard column AG17C 2 × 50 mm and separation column AS17C 2 × 250 mm (Thermo Scientific, USA) were used. A KOH gradient was applied starting with 10 mM for 1 min followed by a linear increase to 50 mM over 4.5 min. Afterwards, the concentration declined over 1 min to 10 mM and it was retained for an additional min before the next measurement. Chromatograms were processed with Chromeleon 6.70 software (Dionex, Thermo Scientific, USA) . Sulfide was determined using themethylene-blue-method with dimethylene-*p*-phenylendiamine and ferric iron solutions [54]. Samples were measured at 670 nm (Biochrom Novaspec 4049 Spectrophotometer, Cambridge, England).

## 3. Results

### 3.1. Sor Activity in Sb. Thermosulfidooxidans

**Figure 1:**
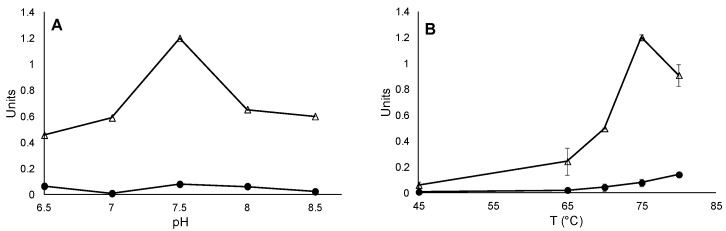
Determination of *Sb. thermosulfidooxidans* Sor properties in crude extracts. Optimal pH values (**A**); and temperature (**B**) were determined for the oxygenase (triangles) and reductase (circles) enzyme activities. In (**A**) experiments were done at 75 °C; and in (**B**) at pH 8. Standard deviation values from triplicates are shown.

After observing the presence of *sor* genes encoded in genomes of sulfobacilli, we measured Sor activity in crude extracts of *Sb. thermosulfidooxidans.* Optimum pH and temperature values for Sor activity were determined. It showed the highest specific oxygenase activity (1.2 U/mg) at 75 °C and pH 7.5. The reductase activity at this condition was 77 mU/mg (Figure 1). Interestingly, a higher reductase activity (140 mU/mg) was measured at 80 °C. The optimum conditions for reductase activity were not determined since enzyme activities at higher temperatures were not analyzed. Neither oxygenase nor reductase activities were found when *Sb. thermosulfidooxidans* cells grown on ferrous iron were analyzed, suggesting the presence of possible regulatory mechanisms controlling Sor expression (not shown). To validate our assays, we measured *S. metallicus* Sor as positive control. Although Sor enzyme activity has not been characterized earlier in this archaeon, the presence of *sor* gene transcripts has been reported. Higher levels of expression were found in S° grown cells, compared to iron and pyrite grown ones [55]. At its optimal growth temperature (65 °C), a specific oxygenase activity of 0.22 U/mg and a specific reductase activity of 50 nU/mg were measured in crude extracts. No detailed parameters for *S. metallicus* optimum temperature or pH were determined since it was not the main goal of our study.

### 3.2. Genes Probably Involved in RISC Metabolism of Sb. Thermosulfidooxidans

*Sb. thermosulfidooxidans* is able to oxidize S°, thiosulfate and tetrathionate [56]. The sequences of proteins involved in RISC oxidation in *At. ferrooxidans* [36] and *At. caldus* [24] were used to search for homologous genes encoded in the *Sb. thermosulfidooxidans* genome database. Several genes likely to be involved in RISC oxidation were found. Among them, two *sor* genes (Sulth_1627 and Sulth_1798) and one complete cluster of *hdr* genes (Sulth_1021-Sulth_1026) were found. The *doxD* component of the Tqo (Sulth_1689), three putative *sqr* (Sulth_0548; Sulth_0580 and Sulth_0946) and three putative *tth* genes (Sulth_0921; Sulth_1188; Sulth_3251) were identified as well (Table 2). Contrary to *At. caldus*, *doxD* and *tth* were not found clustered in *Sb. thermosulfidooxidans.* Several genes encoding proteins with a rhodanese domain were also found. Interestingly, no *sox* genes were found.

### 3.3. Does At. caldus Possess an Active Sor Enzyme?

**Figure 2:**
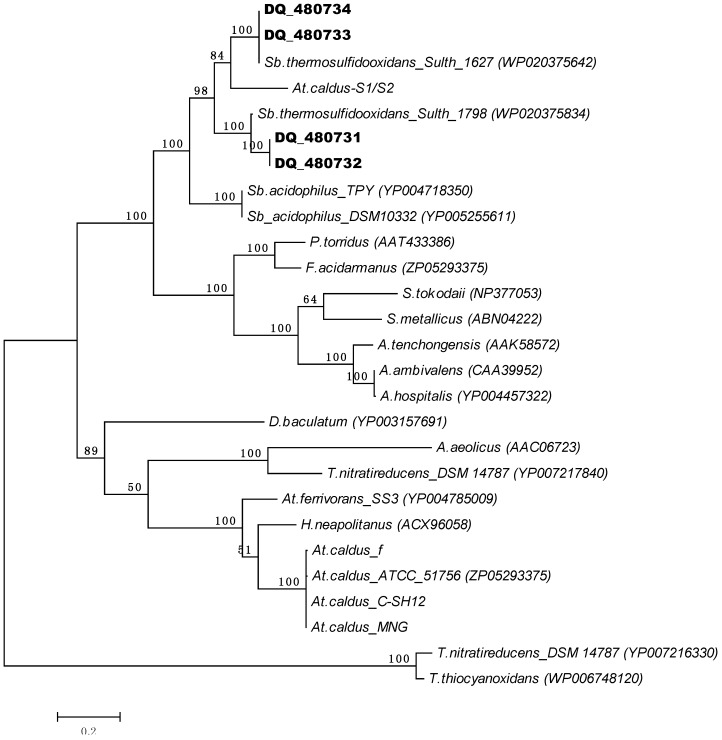
Maximum likelihood phylogenetic tree showing relationship amongst bacterial and archaeal Sor proteins. Sor aminoacidic sequences of *Sb. thermosulfidooxidans* DSM 9293^T^ (WP_020375642 and WP-_020375834), *A. aeolicus* VF5 (NP_21332), *H. neapolitanus* C2 (YP_003263105), *At. ferr*ivorans SS3 (YP_004785009), *At. caldus* ATCC 51756; DSM 8589 (ZP_05293375), *A. tengchongensis* (AAK58572), *A. ambivalens* (CAA39952), *Acidianus hospitalis*(YP_004457322), *Sulfolobus tokodaii* (NP_377053), *Picrophilus torridus* (AAT43386), *Ferroplasma acidarman*us fer1 (ZP_01708456), *Desulfomicrobium baculatum* DSM 4028 (YP_003157691), *Sb. acidophilus* DSM 10332^T^ (YP_005255611), *Sb. acidophilus* TPY (YP_004718350), *S. metallicus* (ABN04222), *Thioalkalivibrio nitratireducens* DSM14787_1 (YP_007217840), and DSM14787_2 (YP_007216330), *Thioalkalivibrio thiocyanoxidans* (WP_006748120) were used. Additionally, Sor sequences from four metagenomic clones (DQ480731, DQ480732, DQ480733/ ABF20540, DQ48074/ABF20541) [41] and the SOR sequences obtained from *At. caldus* strains MNG, C-SH12, f and S1 and S2, obtained in this study (see text), were included.

Previously, we had reported Sor activity in *At. caldus* S1 and S2 strains [40]. However, after several attempts we could not detect Sor activity in any of the seven *At. caldus* strains studied. Additionally, no *sor* gene is encoded in the genome sequence of strain SM-1 [13]. To answer the question of how conserved the *sor* gene is in *At. caldus*, we screened seven strains by PCR with CODEHOP primers designed based on alignments of bacterial Sor sequences. Positive *sor* gene amplicons were detected in six of them. These amplicons (~800 bp), representing 80% of the complete Sor protein, were cloned and sequenced. A phylogenetic tree showed that these *At. caldus* Sor sequences clustered within the acidithiobacilli branch (Figure 2), which also includes the Sor from *H. neapolitanus* and *At. ferrivorans* SS3 [34]. Surprisingly, the Sor aminoacid sequences obtained from *At. caldus* S1 and S2 strains, which are 100% identical (shown as S1/S2), clustered within the S*ulfobacillus* Sor branch (Figure 2). In this context, it is highly probable that our previously reported Sor activity in these strains was due to the presence of a *Sulfobacillus* contaminant. The purity of these cultures was checked by nested PCR using 16S rDNA primers for *Sulfobacillus* and *Acidithiobacillus*, confirming the presence of *Sulfobacillus* (Figure S1). Sequence analysis of the obtained *sor* amplicon from the *Sulfobacillus* contaminant strain revealed a high similarity with *Sulfobacillus* L15 (data not shown). Further PCR tests were done to discard the presence of *At. thiooxidans*, *At. ferrooxidans*, *Leptospirillum sp.* and *archaea* in S1 and S2 strains. After re-purification of *At. caldus* S1 and S2 strains in our laboratory, their *sor* gene sequences were 100% identical to the *At. caldus*^T^ (not shown). Nevertheless, no Sor activity was detected in S° grown cells under our assay conditions.

**Table 2:** Proteins related to sulfur metabolism encoded in *Sb. thermosulfidooxidans* genome.

Locus_Tag	Protein Annotation	Homologous in *At. caldus*	BlastP Identity
Sulth_0548	FAD-dependent pyridine nucleotide-disulfideoxidoreductase	Sqr_1 (WP_004871912)	65%
Sulth_0580	FAD-dependentpyridinenucleotide-disulfideoxidoreductase	Sqr_1 (WP_004871912)	58%
Sulth_0921	Pyrrolo-quinolinequinone repeat-containing protein	Tetrathionate hydrolase WP_004873216.1	40%
Sulth_0946	FAD-dependentpyridinenucleotide-disulfideoxidoreductase	Sulfidequinoneoxidorreductase Sqr_1 (WP_004871912)	62%
Sulth_1021	Heterodisulfidereductase, subunit C	Heterodisulfidereductase, subunit C HdrC (WP_038472248.1)	52%
Sulth_1022	Heterodisulfidereductase, subunit B	Heterodisulfidereductase, subunit B HdrB (WP_051620817.1)	59%
Sulth_1023	FAD-dependent pyridine nucleotide-disulphide oxidoreductase	pyridinenucleotide-disulfideoxidoreductase (WP_004868630.1)	41%
Sulth_1024	Hypotheticalprotein	Hypotheticalprotein (WP_004868631.1)	30%
Sulth_1025	Iron-sulfur cluster-binding protein	Heterodisulfidereductase, subunit C HdrC(WP_004868632.1)	32%
Sulth_1026	unknown function DUF224 cysteine-rich region domain protein	Heterodisulfidereductase, subunitB HdrB (WP_004868633.1)	38%
Sulth_1046	DsrEfamilyprotein	Disulfidereductase(WP_004868633.1)	31%
Sulth_1188	Pyrrolo-quinolinequinone repeat-containing protein	Tetrathionate hydrolase (WP_004873216.1)	31%
Sulth_1355	Adenylyl-sulfate kinase	Adenylyl sulfate kinase (WP_004868315.1)	40%
Sulth_1366	Sulfate adenylyltransferrase	Adenylyl sulfate kinase (WP_004868315.1)	39%
Sulth_1433	Sulfate adenylyltransferrase	Adenylyl sulfate kinase (WP_004868315.1)	38%
Sulth_1435	Sulfate adenylyltransferrase	Adenylyl sulfate kinase (WP_004868315.1)	44%
Sulth_1627	Sulfuroxygenasereductase	Sulfuroxygenasereductase (WP_004871908.1)	48%
Sulth_1680	Rhodanese like protein	Sulfur transferase(WP_004872361.1)	32%
Sulth_1689	Tqo small subunit DoxD domain-containing	Quinol oxidase (WP_004873215.1)	34%
Sulth_1798	Sulfuroxygenasereductase	Sulfur transferase(WP_004872361.1)	47%
Sulth_1878	Rhodanese-likeprotein	Sulfurtransferase(WP_004872361.1)	29%
Sulth_2335	Rhodanese-likeprotein	Sulfurtransferase(WP_004868554.1)	35%
Sulth_2366	Nitratereductase	Formate dehydrogenase (WP_004868564.1)	50%
Sulth_2367	Sulfur reductase beta subunit	Ferredoxin (WP_004868562.1)	55%
Sulth_2368	DMSO reductase anchor subunit	dimethyl sulfoxidereductase subunit C (WP_004872154.1)	27%
Sulth_2770	Heterodisulfidereductase, subunit C	Heterodisulfidereductasesubunit C (WP_038472248.1)	47%
Sulth_2771	Heterodisulfidereductase, subunit B	Heterodisulfidereductasesubunit B (WP_051620815.1)	50%
Sulth_2772	FAD-dependent pyridine nucleotide-disulphide oxidoreductase	Pyridinenucleotide-disulfideoxidoreductase(WP_004868887.1)	42%
Sulth_3040	Rhodanese-likeprotein	Sulfurtransferase(WP_004872361.1)	30%
Sulth_3251	Pyrrolo-quinolinequinone repeat-containing protein	Tetrathionate hydrolase (WP_004873216.1)	54%
Sulth_3294	Rhodanese-likeprotein	Sulfurtransferase(WP_004872361.1)	31%

## 4. Discussion

*Sb. thermosulfidooxidans* crude extracts possess an active Sor enzyme. Our results are in agreement with the “thermophilic” nature of Sor. The recombinant Sor from *H. neapolitanus* was shown to be active in a temperature range of 10–99 °C with an optimum at 80 °C [20]. In *Sb. thermosulfidooxidans* Sor reaction products such as sulfite, thiosulfate and sulfide can be further metabolized and coupled with energy conservation by enzymes such as Saor, Tqo, Tth and Sqr, which have been found to be encoded in its genome sequence. The low reductase activities measured may be related to (i) the utilization of crude extracts, in which the presence of enzymes such as Sqr may contribute to their degradation; and (ii) some hydrogen sulfide loss prior to its fixation. Since two *sor* genes were found, further research is needed to elucidate their regulation and their connection with other proteins likely involved in RISC oxidation in *Sb. thermosulfidooxidans*.

Several proteins with a Rhodanese domain were found to be encoded in the *Sb. thermosulfidooxidans* genome sequence. These may contribute to the oxidation of persulfides or polysulfides by acting as sulfur transferases [39]. This bacterium also possesses the *hdr* gene cluster, which could also be responsible for S° oxidation in *At. ferrooxidans* as well as some other acidophiles [36]. Considering this, in *Sb. thermosulfidooxidans*, S° produced from hydrolysis of tetrathionate by Tth or oxidation of H_2_S by Sqr could be accumulated in the form of polysulfides, which after being transferred into the cytoplasm, can be further oxidized via Sor or Hdr. Although no biochemical evidence for involvement of the Hdr complex in S° oxidation in acidophiles has been demonstrated yet, we recently found several Hdr proteins expressed by shotgun proteomics of *At. ferrooxidans* ATCC 23270^T^ biofilm formation process on pyrite, [57]. Recently, a comparison was done among isolates and environmental *Sulfobacillus* genomes. For this, five new draft genomes of *Sulfobacillus* spp. were assembled from metagenomic data obtained from the Iron Mountain, California. These sequences were compared with *Sb. acidophilus* TPY [8] and *Sb. thermosulfidooxidans* Cutipay [6]. The analysis showed the presence of *sor* genes in two of the five genomes assembled, while one Hdr cluster was found in all of them [58].

Chen *et al.* reported four *sor* sequences obtained from metagenomic DNA samples from a bioreactor containing *Leptospirillum*, *Sulfobacillus*, *Acidithiobacillus* and *Sphingomonas spp.* One *sor* gene sequence was cloned and expressed in *E. coli* and the recombinant Sor was active [41]. Due to increased amounts of genomic information we reanalyzed these four *sor* sequences (Genbank accession numbers DQ480731–DQ480734) by Blast in the JGI database. These Sor proteins clustered within *Sulfobacillus* Sor proteins, showing 99%–100% identities with *Sulfobacillus* sequences (Sulth_1627 and Sulth_1798) and 44%–48% with *At. caldus* sequences (Figure 2). These results, plus the absence of a *sor* gene in *the At. caldus* SM-1 genome sequence [13], strongly suggest that a part of the *Sulfobacillus sor* gene was cloned and attributed to belong to *At. caldus* SM-1.

Whether Sor contributes to the overall sulfur oxidation in *At. caldus* is, in our opinion, still an open question. Sor enzymes contain a mononuclear non-Heme iron site as the putative redox-active cofactor [17]. By site directed mutagenesis it has been shown that the three Fe coordinating residues H86, H90 and E114 as well as the C31 (in *A. ambivalens* numbering), are essential for catalysis [59]. Analysis of *At. caldus* Sor sequence shows conservation of all of the residues relevant for the coordination of iron as well as the C31 (Supplementary Figure S2). Several strains possess a *sor* gene but to the best of our knowledge, its enzyme activity has not been successfully measured in any *At. caldus* strain, neither in crude extracts nor cell fractions. No significant differences on the levels of *sor* transcripts were reported between *At. caldus*^T^ cells grown with tetrathionate or S° as electron donors [24]. In the same study, no protein spot could be identified as Sor in a two dimensional polyacrilamyde gel electrophoresis (2D-PAGE). We have also measured very low levels of the *sor* gene transcript by Real time reverse transcription (RT)-PCR in *At. caldus*^T^, and no significant differences were found when cells grown with S° or thiosulfate as energy sources were analyzed (not shown). Recently, by high throughput proteomics we detected >1300 proteins from sulfur and thiosulfate *At. caldus* grown cells. The Sor protein, encoded by the gene ACA_0302, was not detected in any sample from both growth conditions [60]. A proteomic study of the response of *At. caldus* towards suboptimal pH conditions showed that several proteins involved in sulfur oxidation such as HdrABC and Sqr were induced when cells were incubated at pH 1.1 [61]. Although Sor was not detected in this study, a test of Sor activity in *At. caldus* at acid pH range might be helpful to completely elucidate the presence of Sor activity in this bacterium. Recently, a *sor* mutant of *At. caldus* MTH-04 strain was produced and a differential gene expression study was done by microarrays. No obvious differences were observed in the growth of the *sor* mutant and the wild type strain in media with S° as energy source [62]. However, since enzyme activities were not measured in this study, the question whether Sor was active or not in wild type *At. caldus* cells remains open.

## 5. Conclusions

In this study we provide evidence that *Sb. thermosulfidooxidans* possess Sor activity and that the previously Sor activity reported in *At. caldus* strains S1 and S2 most likely was due to the presence of a *Sulfobacillus* contaminant.

## Supplementary Files

Supplementary File 1
