# Supplementary material for: Sulfur Oxygenase Reductase (Sor) in the Moderately Thermoacidophilic Leaching Bacteria: Studies in Sulfobacillus thermosulfidooxidans and Acidithiobacillus caldus
Source: Microorganisms. 2015 Oct 21;3(4):707–24. doi: 10.3390/microorganisms3040707 (PMC5023260; doi:10.3390/microorganisms3040707)
Supplement: Supplementary File 1 [file microorganisms-03-00707-s001.docx]

Supplementary Material

Sulfur Oxygenase Reductase (Sor) in the Moderately Thermoacidophilic Leaching Bacteria: Studies in
*Sulfobacillus thermosulfidooxidans* and *Acidithiobacillus caldus*

Claudia Janosch ^1^, Francisco Remonsellez ^2^, Wolfgang Sand ^1^and Mario Vera ^1,^*

^1^ Biofilm Centre, Aquatische Biotechnologie, Universität Duisburg-Essen, Universitätstraße5,
Essen 45141, Germany; E-Mails: claudia.janosch@gmx.de (C.J.); fremonse@ucn.cl (W.S.)

^2^ Laboratorio de Tecnología de Membranas, Biotecnología y Medio Ambiente,
Departamento de Ingeniería Química, Universidad Católica del Norte, Antofagasta 1270709, Chile;
E-Mail: fremonse@ucn.cl

***** Author to whom correspondence should be addressed; E-Mail: mario.vera@uni-due.de;
Tel.: +49-201-1837083.


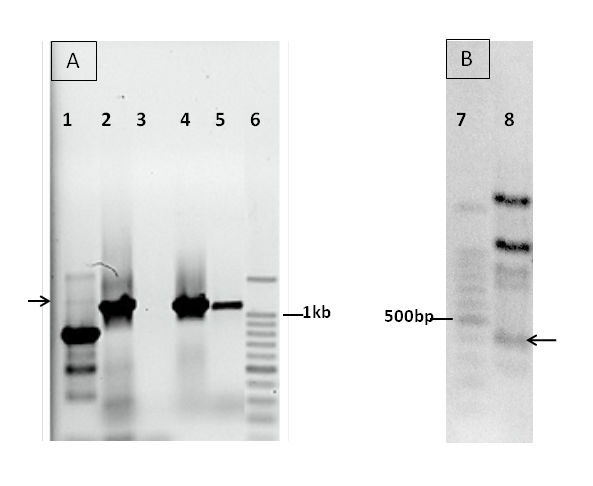


**Figure S1.** PCR detection of a *Sulfobacillus* contaminant in *At. caldus* strain S1; (**A**) Nested PCR with *Sulfobacillus spp.* and *At. caldus* primers for 16S rDNA; **1**. PCR amplicons of
*At. caldus* S1 with primer EUB27F-SULFO1137R; **2**. PCR amplicons of *Sb. thermosulfidooxidans* with primer EUB27F-SULFO1137R; **3**. Negative control with primers EUB27F-SULFO1137R, for *Sulfobacillus*; **4**. PCR amplicons of *At. caldus* S1 with primer CALD460F-EUB1429R; **5**. PCR amplicons of the *At. caldus* type strain with primers CALD460F-EUB1429R;
**6**. 100bp Marker (Promega**^®^**); (**B**) Nested PCR with *Sulfobacillus spp.* primers for 16S rDNA; **7**. 100bp Marker (Promega^®^); **8**. PCR amplicons of *At. caldus* S1 using the PCR reaction from A1 as template, primers SULFO170F-SULFO606R.


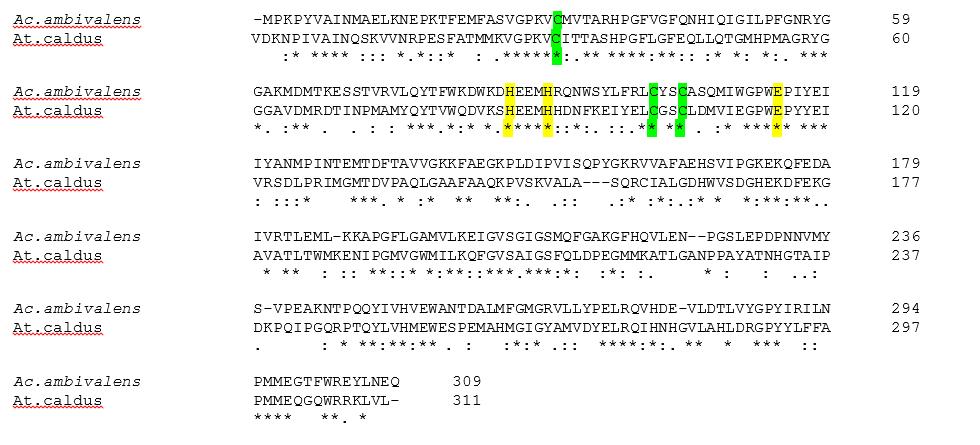


**Figure S2.** Alignment of Sor sequences of *Acidianus ambvivalens* DSM 3772 and *Acidithiobacillus caldus* DSM 8584. The aminoacids involved in the iron binding site are highlighted in yellow, while the three conserved cystein residues present in the active site are highlighted in green.
